# The Human Mesenchymal Stromal Cell-Derived Osteocyte Capacity to Modulate Dendritic Cell Functions Is Strictly Dependent on the Culture System

**DOI:** 10.1155/2015/526195

**Published:** 2015-07-12

**Authors:** Sara Trabanelli, Federico La Manna, Marco Romano, Valentina Salvestrini, Michele Cavo, Marilena Ciciarello, Roberto M. Lemoli, Antonio Curti

**Affiliations:** ^1^Department of Experimental, Diagnostic and Specialty Medicine, Institute of Hematology “L. & A. Seràgnoli”, University of Bologna, 40138 Bologna, Italy; ^2^Ludwig Center for Cancer Research, The University of Lausanne, 1011 Lausanne, Switzerland; ^3^Osteoncology and Rare Tumors Center, Istituto Scientifico Romagnolo per lo Studio e la Cura dei Tumori (IRST) IRCCS, 4714 Meldola, Italy; ^4^MRC Centre for Transplantation, Division of Transplantation Immunology & Mucosal Biology, King's College London, London WC2R 2LS, UK; ^5^Department of Internal Medicine (DiMI), University of Genoa and IRCCS Azienda Ospedaliera Universitaria S. Martino-IST, 16132 Genoa, Italy

## Abstract

*In vitro* differentiation of mesenchymal stromal cells (MSC) into osteocytes (human differentiated osteogenic cells, hDOC) before implantation has been proposed to optimize bone regeneration. However, a deep characterization of the immunological properties of DOC, including their effect on dendritic cell (DC) function, is not available. DOC can be used either as cellular suspension (detached, Det-DOC) or as adherent cells implanted on scaffolds (adherent, Adh-DOC). By mimicking *in vitro* these two different routes of administration, we show that both Det-DOC and Adh-DOC can modulate DC functions. Specifically, the weak downregulation of CD80 and CD86 caused by Det-DOC on DC surface results in a weak modulation of DC functions, which indeed retain a high capacity to induce T-cell proliferation and to generate CD4^+^CD25^+^Foxp3^+^ T cells. Moreover, Det-DOC enhance the DC capacity to differentiate CD4^+^CD161^+^CD196^+^ Th17-cells by upregulating IL-6 secretion. Conversely, Adh-DOC strongly suppress DC functions by a profound downregulation of CD80 and CD86 on DC as well as by the inhibition of TGF-*β* production. In conclusion, we demonstrate that different types of DOC cell preparation may have a different impact on the modulation of the host immune system. This finding may have relevant implications for the design of cell-based tissue-engineering strategies.

## 1. Introduction

MSC are multipotent cells, capable of differentiating,* in vitro,* into different lineages, including osteocytes, chondrocytes, adipocytes, muscle cells, cardiomyocytes, and neural precursor [1]. More recently, the capacity of human MSC (hMSC) to suppress both innate and adaptive immunity has been described [2, 3] as well as their poor immunogenicity. As a result, the therapeutic potential of hMSC as immunoregulatory agents is currently being explored in several phase I/II clinical trials [4–6].

A number of recent studies have focused on the influence of hMSC on DC functions [2, 7, 8]. DC play a critical role in initiating and regulating immune responses [9].* In vitro* DC can be generated from CD34^+^ stem/progenitor cells and from CD14^+^ monocytes [10, 11]. The* in vitro* interaction between hMSC and either CD34^+^ or CD14^+^ DC progenitors inhibits the generation of functional DC [2, 7, 8], skewing their differentiation toward phenotypically abnormal DC, which express lower level of CD1a, CD40, CD80, CD86, and CD83. Moreover, they show an impaired capacity of stimulating allogeneic T cell proliferation. Different mechanisms are responsible for the effects of hMSC on DC differentiation including the involvement of both soluble factors [8] and cell-to-cell contact interactions [12, 13]. Taken together, these studies demonstrated the profound immunosuppressive effects of hMSC on DC. However, the topic of whether the immunological properties of hMSC persist after* in vitro* differentiation into hDOC has been not deeply investigated. Indeed, it has only been shown that hDOC suppress T cell proliferation elicited by allogeneic cells [14, 15]. In fact, the characterization of the immunological properties of hDOC may be of crucial importance for cell-based tissue-engineering therapeutic strategies. Although hMSC have been shown to contribute to repair bone defects* in vivo* either through infusion or local implantation [16, 17], more recent strategies aim to* in vitro* differentiate hMSC into hDOC before implantation, in order to optimize bone regeneration [18, 19]. Therefore, the evaluation of the interactions between hDOC and the host allogeneic immune system, in particular of antigen presenting cells (APC) such as DC, may be important in view of clinical trials. Since the preparation of hDOC for cell-based tissue-engineering strategies may be different [18, 19], it is interesting to evaluate whether different culture systems may affect the capacity of DOC of modulating the immune response. For these reasons, here we characterized the immunological properties of hDOC manipulated in two different ways, which mimic the use of hDOC as cell suspension or as adherent cells, by showing their capacity to modulate the phenotype and the functions of allogeneic DC.

## 2. Materials and Methods

### 2.1. Osteogenic Differentiation of hMSCs

Human MSCs (hMSCs) were isolated from BM aspirates of healthy donors after obtaining written informed consent. The characteristics of the donors are shown in Table S1 (see Supplementary Material available online at http://dx.doi.org/10.1155/2015/526195). To induce hDOC, hMSCs were seeded at 3.1 × 10^3^ cells/cm^2^ and grown in osteogenic differentiation medium (Lonza) containing L-glutamine, MCGS, dexamethasone, ascorbate, *β*-glycerophosphate, and penicillin/streptomycin [20]. Medium was replaced every 3-4 days for 2 weeks. The osteogenic differentiation was analysed by cytological staining and by the evaluation of RUNX2 mRNA levels (Figure S1). Calcium deposition was determined by using Alizarin red staining. Briefly, cells were fixed in 10% PFA in PBS for 15 min at room temperature (RT), rinsed with PBS and distilled water, and then stained with 40 mM Alizarin red solution (Sigma Aldrich) pH 4.2, for 75 min at RT with gentle agitation. After washing, Alizarin red was extracted from fixed cells by incubating in 10% (w/v) cetylpyridinium chloride (CPC) solution (Sigma Aldrich) in 10 mM sodium phosphate for 15 min at RT with gentle agitation. After 2 weeks hMSC and hDOC were lysed and total RNA was extracted using RNeasy Micro kit (Qiagen) and reverse transcribed using a Promega ImProm II kit and random examers in 20 *μ*L final volume. Quantitative real-time PCR was performed using an ABI Prism 7900 sequence detection system (Applied Biosystems). Quantitative real-time PCR data were analysed using the 2^−ΔΔCt^ method. The relative level of RUNX2 mRNA was calculated by subtracting Ct values of the control gene (GAPDH) from the Ct values of the specific gene (RUNX2). Undifferentiated hMSC were used as reference and taken as value of 1. Primers probe for RUNX2, Hs00231692_m1, and GAPDH, Hs00266705_g1 were purchased from Applied Biosystems.

### 2.2. Cell Plating

For immunological assays, hDOC were used either in adhesion on the same plate used for differentiation (Adh-DOC) or after harvesting and replating (Det-DOC). For harvesting, monolayers of hDOC were washed with washing buffer (0.5% BSA, 5 mM EDTA in PBS), incubated with trypsin (0.25% trypsin with 0.1% EDTA, EuroClone), and collected with a cell scraper. Where indicated the cellular component was lysed by incubating the monolayer with 0.1 M NaCl, 0.01 M TRIS, 0.2% EDTA, and 0.1% TritonX-100.

In all the assays, the same number of Adh-DOC and Det-DOC was used. For each experiment, the cell number was evaluated the day before the assays, by counting 3 wells of Adh-DOC harvested separately. The mean values of these 3 counts were used to plate Det-DOC. Then, Adh-DOC and Det-DOC were cultured overnight in complete medium (RPMI 1640 medium (Lonza) supplemented with 10% heat-inactivated FBS (Gibco-Invitrogen), 2 mM L-glutamine, 100 U/mL penicillin, and 100 *μ*g/mL streptomycin (MP Biomedicals)) at 37°C in 5% CO_2_, before coculturing with DC and CD3^+^ T cells. The experimental strategy is showed in a schema (Figure S2). The same strategy and the same conditions were used to plate Det-MSC. Indeed, the number of Det-MSC to plate was established by counting independently 3 wells of Adh-MSC.

### 2.3. Cell Isolation and DC Generation

Buffy coats were obtained from healthy donors and were used to isolate mononuclear cells (MNC), by gradient centrifugation (Lymphoprep; 1.077 g/mL; Nycomed Pharma). After separation, CD14^+^ monocytes and CD3^+^ T cells were purified from total MNC by magnetic separation columns (Miltenyi Biotec), according to manufacturer's instructions. The characteristics of the donors are shown in Supplementary Materials (Table S1). Monocyte-derived DCs (Mo-DCs) were generated by a 5-day culture of CD14^+^ cells in complete medium plus 50 ng/mL granulocyte-macrophage colony-stimulation factor (GM-CSF) and 800 U/mL IL-4 (both by Endogen), at 37°C in 5% CO_2_. For maturation, day 5 Mo-DCs were cultured with GM-CSF and IL-4 and incubated for 48 hours in presence of a cocktail of cytokine made of 10 ng/mL TNF*α*, 10 ng/mL IL-6, 10 ng/mL IL-1*β*, and 1 *μ*g/mL PGE_2_ (all by Endogen) [21].

### 2.4. Mixed Leukocyte Reaction (MLR)

To detect lymphocytes proliferation, CD3^+^ T cells were labelled with carboxyfluorescein succinimidyl ester (CFSE, 2.5 *μ*M from Sigma Aldrich) before plating [22]. To test the capacity of Adh-DOC and Det-DOC to modulate the DC allostimulatory capacity, allogeneic Mo-DC, either immature or mature, were irradiated (3000 cGy) and plated (1 : 1) at 37°C on either Adh-DOC or Det-DOC in presence of third party CFSE-labeled CD3^+^ T cells (1 : 10). As a positive control, CD3^+^ T cells were cultured with Mo-DC and, as a negative control, with medium alone. For a second set of experiments, CD3^+^ T cells were added to the upper chamber of a 0.4 *μ*m pore polycarbonate filter in 24-well transwell chambers (Corning Costar) to keep them separated from DC and from either Adh-DOC or Det-DOC, which were plated in the lower chamber of the transwell system. After 5 days, the cells in the upper chamber were collected and analyzed using BD FACSCantoII equipment (BD Biosciences).

### 2.5. Induction of CD4^+^CD25^+^Foxp3^+^ and of CD4^+^CD161^+^CD196^+^ T Cells

To test the capacity of Adh-DOC and Det-DOC to modulate the DC capacity to induce in CD3^+^ T cells the T_reg_ cell or the Th17 cell phenotype, allogeneic Mo-DC, either immature or mature, were plated (1 : 1) on either Adh-DOC or Det-DOC in presence of allogeneic CD3^+^ T cells (1 : 20). As a positive control, CD3^+^ T cells were cultured with Mo-DC, as a negative control, with medium alone [23]. For a second set of experiments, CD3^+^ T cells were added to the upper chamber of a 0.4 *μ*m pore polycarbonate filter in 24-well transwell chambers (Corning Costar), while DC and either Adh-DOC or Det-DOC were added in the lower chamber.

Cell cultures were incubated at 37°C for 5 days; then the T cells were harvested from the upper chamber and stained for immunophenotype via tricolor immunofluorescence, which was performed using fluorescein isothiocyanate- (FITC-) conjugated anti-human CD4 (clone RPA-T4), phycoerythrin- (PE-) conjugated anti-human Foxp3 (clone 206D), and allophycocyanin- (APC-) conjugated anti-human CD25 (clone BC96, Biolegend). For cell-surface staining, 1 × 10^5^ cells/100 *μ*L were incubated in the dark for 20 min at RT with mAbs in phosphate-buffered saline- (PBS-) 1% bovine serum albumin. Subsequently, for Foxp3 intracellular staining, cells were incubated at RT in the dark for 20 min with fix/perm buffer followed by 15 min with perm solution and additional 30 min with the mAb. After 2 washes, samples were analyzed using BD FACSCantoII equipment (BD Biosciences). A minimum of 10,000 events was collected in list mode on FACSDiva software.

### 2.6. Mo-DC Phenotype

Immature and mature Mo-DC were incubated for 5 days either alone or with Adh-DOC or Det-DOC (1 : 1). Dual-color immunofluorescence was performed using the following panel of mAbs: PE- or FITC-conjugated anti-human HLA-DR (BD Pharmingen; clone L242), CD86 (Biolegend; clone IT2.2), and CD80 (Biolegend; clone 2D10). Negative controls were isotype-matched irrelevant mAbs. Cells were analyzed by using BD FACSCantoII equipment (BD Biosciences). A minimum of 10,000 events was collected in list mode on FACSDiva software.

### 2.7. Cytokine Production

Immature and mature Mo-DC were incubated for 5 days either alone or with Adh-DOC or Det-DOC (1 : 1). Supernatants were collected and tested for the release of TGF-*β*1 (DRG Diagnostics, Marburg, Germany) IL-6 and IL-10 (Thermo Scientific, Erembodegem, Belgium), according to the manufacturer's instructions.

### 2.8. Statistical Analysis

Data were analyzed using FlowJo software (TreeStar). Results are expressed as mean ± SEM. Statistical analysis was performed using the Kruskal-Wallis test. *∗* = *p* < 0.05, *∗∗* = *p* < 0.01, and *∗∗∗* = *p* < 0.001, Bonferroni corrected.

## 3. Results

### 3.1. hDOC Suppress DC-Induced T Cell Proliferation

**Figure 1:**
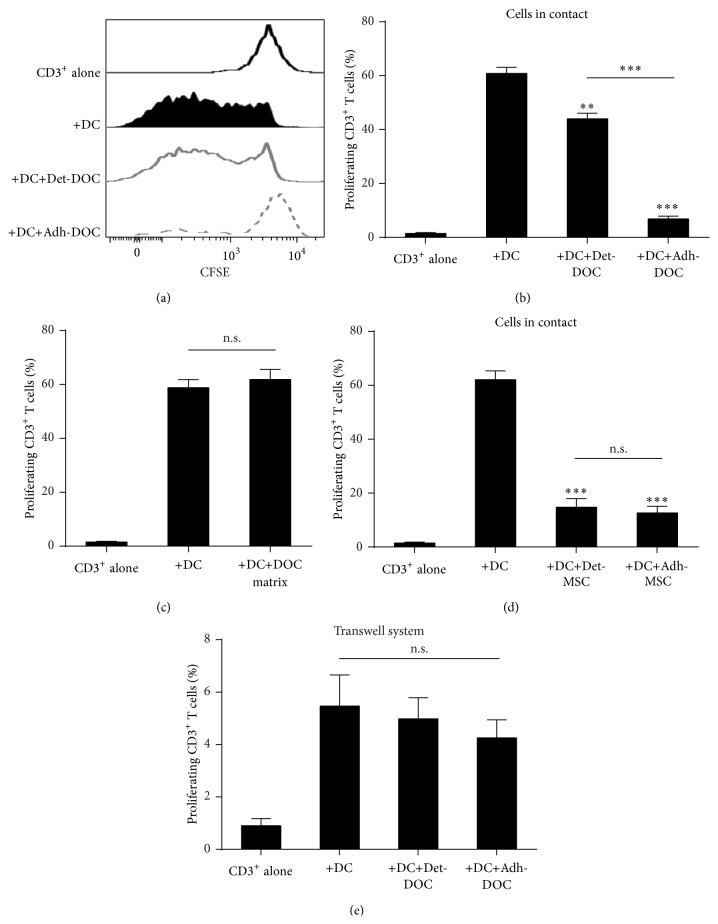
Modulation of DC-induced T cell proliferation by hDOC. CD3^+^ T cells were cultured for 5 days either alone (negative control) or with allogeneic immature DC (positive control, +DC) or with both DC and hDOC. To evaluate the effect of detachment on the immunomodulatory function of hDOC, hDOC were either assayed with CD3^+^ and DC after detachment (+DC+Det-DOC) or left in adhesion (+DC+Adh-DOC). The ratio of seeded cells in coculture was the following: CD3^+^ T cell : DC : DOC = 10 : 1 : 1. (a) CFSE staining of CD3^+^ T cell alone (black line), CD3^+^ T cells with DC (black filled), CD3^+^ T cells with DC in presence of either Det-DOC (grey line), or Adh-DOC (dashed grey line); (b) histograms show the percentage of proliferating CD3^+^ T cells under the different conditions. (c) Effects of hDOC-derived extracellular matrix on CD3^+^ T cells proliferation. CD3^+^ T cells and immature DC were cultured for 5 days on hDOC-derived extracellular matrix, obtained by chemical lysis of Adh-DOC. (d) The same experiment as in (a) and (b) but using undifferentiated hMSC instead of hDOC. (e) The same experiment as in (a) and (b), performed in a 0.4 *μ*m pore polycarbonate transwell system. Upper chamber: CD3^+^ T cells. Lower chamber: DC and hDOC. Histograms represent the mean ± SEM of the percentage of proliferating CD3^+^ T cells of 7 independent experiments. *∗∗* = *p* < 0.01 and *∗∗∗* = *p* < 0.001, Bonferroni corrected.

We first compared the suppressive capacity of hDOC on DC-mediated T cell proliferation. In particular, we used hDOC either after detachment from culture plates (Det-DOC) or as adherent cells (Adh-DOC) to mimic,* in vitro*, the conditions in which they are used therapeutically (i.e., cultured and then detached to be injected as cellular suspension or grown adherent on a scaffold and implanted without detachment [17]). As shown in Figures 1(a) and 1(b), both Det-DOC and Adh-DOC inhibited the allostimulatory capacity of DC (Kruskal Wallis *p* < 0.0001). However, the inhibition induced by Adh-DOC is much stronger than Det-DOC (*p* < 0.001, Bonferroni corrected). Thus, we asked why Det-DOC partially lost their ability to inhibit DC allostimulation.

To investigate the mechanism(s) involved in such process, we first asked whether Det-DOC were less viable than Adh-DOC, since they were harvested after treatment with trypsin and collected with a cell scraper. We could not find a reduction of viability in harvested Det-DOC (data not shown). Then, we asked whether the extracellular matrix secreted by hDOC (routinely discarded to obtain Det-DOC) could itself inhibit the allostimulatory capacity of DC. Therefore, we lysed the cellular component of hDOC and recovered the extracellular matrix to perform the same assay. As shown in Figure 1(c), the extracellular matrix had no inhibitory capacity by itself.

Then, we asked whether the procedure used to detach cells from the plates influenced by itself the tolerogenic capacity of detached DOC. Thus, we compared the capacity of undifferentiated hMSC used after detachment from culture plates (mimicking Det-MSC) with that of cells used as adherent cells (mimicking Adh-MSC). As shown in Figure 1(d), both Det-MSC and Adh-MSC were able to inhibit the DC allostimulation at the same level, suggesting that the detachment procedure, by itself, had no influence on the tolerogenic capacity of the cells.

Finally we investigated whether the different capacity to inhibit DC allostimulation of Det-DOC and Adh-DOC was mediated by soluble factors rather than by a cell-to-cell contact-dependent mechanism(s). Therefore, we performed the same suppression assay by separating CD3^+^ T cells with a 0.4 *μ*m pore transwell chamber, which allows the migration of only soluble factors. As shown in Figure 1(e), neither Det-DOC nor Adh-DOC could inhibit DC allostimulation when DC are cultured without any contact with CD3^+^ T cells. Therefore, in this setting, hDOC modified the allostimulatory capacity of DC, mainly by modulating cell-to-cell contact-dependent mechanism(s).

### 3.2. hDOC Modulate the DC Expression of Costimulatory Molecules

**Figure 2:**
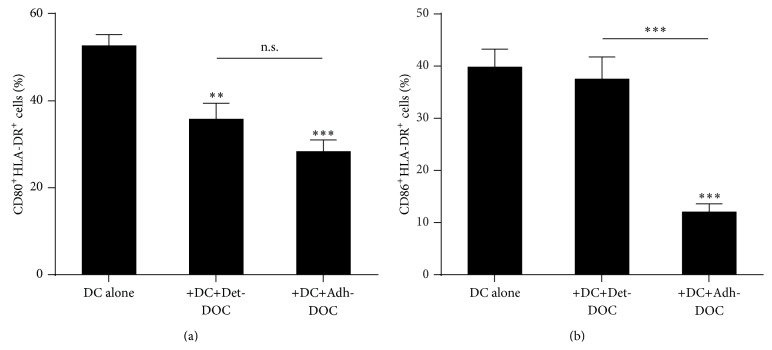
Expression of CD80 and CD86 on DC cocultured with hDOC. Immature DC were cultured for 5 days in the presence or the absence of either Det-DOC or Adh-DOC. The expression of CD80 (a) and CD86 (b) on the HLA-DR^+^ DC was evaluated by flow cytometry. Histograms represent the mean ± SEM of the percentage of DC expressing CD80 and CD86 of 7 independent experiments. *∗∗* = *p* < 0.01 and *∗∗∗* = *p* < 0.001, Bonferroni corrected.

Thus, we wanted to investigate which surface marker(s) on DC may be affected by the inhibitory activity of hDOC [24, 25]. To this end, we cocultured DC with either Det-DOC or Adh-DOC, for 5 days and then analyzed DC phenotype (Kruskal-Wallis *p* < 0.0001 for CD80, *p* = 0.0012 for CD86). To discriminate DC from hDOC we gated on HLA-DR^+^ cells. As shown in Figure 2(a), the coculture of DC with Det-DOC or Adh-DOC resulted in the downregulation of CD80. Notably, Adh-DOC and Det-DOC could downregulate this marker to a comparable extent. Conversely, Det-DOC were not able to downregulate CD86, while this molecule was strongly downregulated by Adh-DOC, as shown in Figure 2(b).

Taken together, our results demonstrate that, if hDOC are left in their own extracellular matrix (Adh-DOC), they acquire a high capacity to inhibit the DC expression of molecules involved in T cell costimulation. On the other hand, if the interactions of hDOC with their extracellular matrix are destroyed (i.e., hDOC are harvested from their own extracellular matrix and seeded back in a new culture support, as for Det-DOC), they partly lose this capacity, resulting in lower inhibition of costimulatory molecules on DC and, consequently, of T cell proliferation.

### 3.3. hDOC Suppress DC-Induced CD4^+^CD25^+^Foxp3^+^ T Cell Differentiation

**Figure 3:**
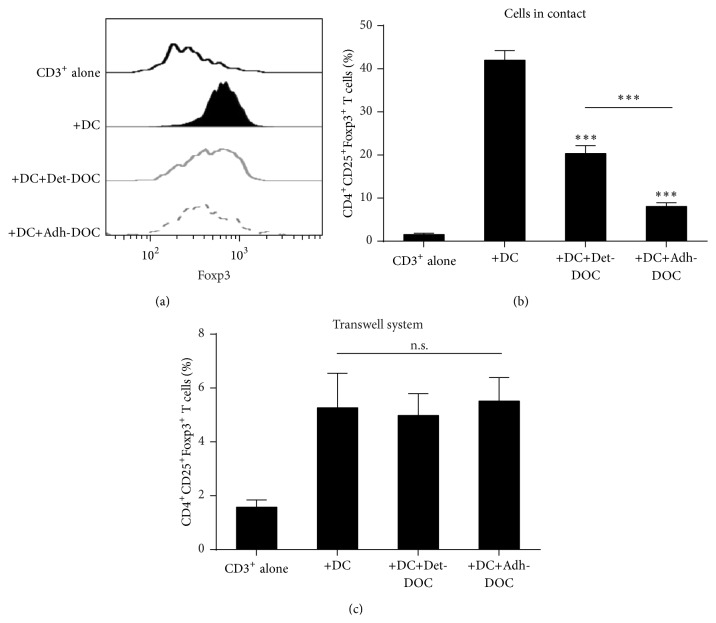
Induction of CD4^+^CD25^+^Foxp3^+^ T cells by DC in the presence of hDOC. CD3^+^ T cells were cultured for 5 days with allogeneic immature DC, alone (positive control), or with either Det-DOC or Adh-DOC Det-DOC or Adh-DOC. The ratios of seeded cells in coculture were the following: CD3^+^ : DC : DOC = 20 : 1 : 1. (a) Foxp3 staining of the CD4^+^CD25^+^ cells fraction within CD3^+^ T cells, either cultured alone (black line) or cultured with DC (black filled), or cultured with DC in presence of either Det-DOC (grey line) or Adh-DOC (dashed grey line). (b) Histograms show the percentage of T cells coexpressing CD4, CD25, and Foxp3. (c) The same experiment as in (a) and (b), performed in a 0.4 *μ*m pore polycarbonate transwell system. Upper chamber: CD3^+^ T cells. Lower chamber: DC and DOC. Histograms represent the mean ± SEM of 7 independent experiments. *∗∗∗* = *p* < 0.001, Bonferroni corrected.

DC have a crucial role not only in the activation of T cell response but also in the induction of tolerance by generating CD4^+^CD25^+^Foxp3^+^ T cell population [26]. Therefore, we asked whether Det-DOC and Adh-DOC could modulate this function of DC. To test this hypothesis, we cocultured DC with allogeneic T cells, in the presence or absence of either Det-DOC or Adh-DOC (Kruskal-Wallis *p* < 0.0001). Coculture of T cells with DC increased the percentage of CD4^+^CD25^+^Foxp3^+^ T cells, as compared to CD3^+^ T cells alone (Figures 3(a) and 3(b)). The addition of Det-DOC or Adh-DOC decreased the population of CD4^+^CD25^+^Foxp3^+^ T cells induced by DC (Figures 3(a) and 3(b)), with the Adh-DOC showing the greatest inhibitory effect (*p* < 0.001, Bonferroni corrected). Then, we asked whether the different capacity to inhibit the DC-mediated CD4^+^CD25^+^Foxp3^+^ T cell generation of Det-DOC and Adh-DOC was mediated by soluble factors rather than by a cell-to-cell contact-dependent mechanism. Therefore, we performed the same assay by separating CD3^+^ T cells with a 0.4 *μ*m pore transwell chamber. As shown in Figure 3(c), Det-DOC and Adh-DOC did not inhibit the capacity of DC to induce a CD4^+^CD25^+^Foxp3^+^ T cell population and there was no significant difference among different hDOC. Therefore, Adh-DOC had an enhanced inhibitory effect on DC to induce CD4^+^CD25^+^Foxp3^+^ T cell population, in comparison to Det-DOC, by modulating mechanism(s) involved in cell-to-cell, contact-dependent suppression. As shown in Figure 2, Adh-DOC were able to downregulate the expression of both CD80 and CD86, while Det-DOC decreased only CD80 at a lower extent. Notably, both these costimulatory molecules are important for the generation of CD4^+^CD25^+^Foxp3^+^ T cells [27].

Taken together, these data demonstrate that hDOC inhibit the capacity of DC to induce CD4^+^CD25^+^Foxp3^+^ T cells. However, such inhibitory capacity is markedly increased when hDOC are left adherent to the same plate used for differentiation (Adh-DOC), whereas it is reduced when hDOC are detached from culture plates (Det-DOC). This effect may be mediated by different modulation of CD80 and CD86 costimulatory molecules on DC by the two different preparations of hDOC.

### 3.4. hDOC Suppress DC-Induced CD4^+^CD161^+^CD196^+^ T Cell Differentiation

**Figure 4:**
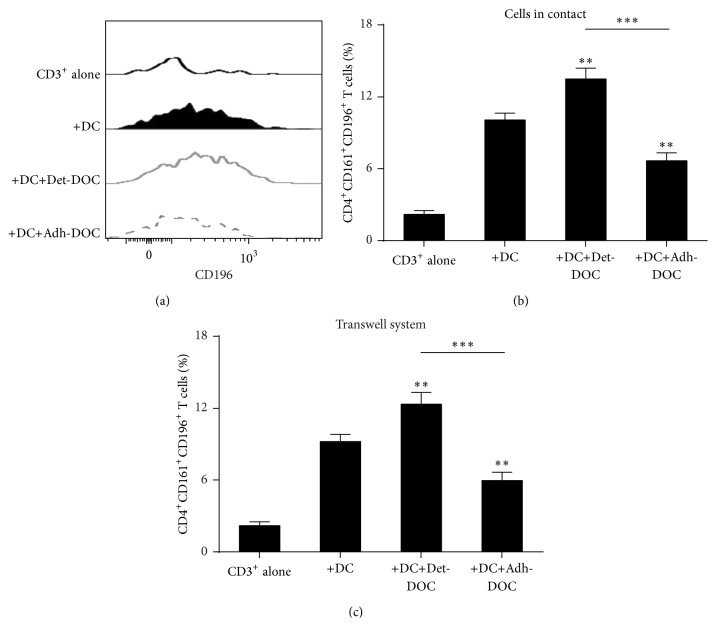
Induction of CD4^+^CD161^+^CD196^+^ T cell by DC in the presence of hDOC. CD3^+^ T cells were cultured for 5 days with allogeneic immature DC, alone (positive control), or with either Det-DOC or Adh-DOC Det-DOC or Adh-DOC. The ratios of seeded cells in coculture were the following: CD3^+^ : DC : DOC = 20 : 1 : 1. (a) CD196 staining of the CD4^+^CD161^+^ cells fraction within CD3^+^ T cells, either cultured alone (black line) or cultured with DC (black filled) or cultured with DC in presence of either Det-DOC (grey line) or Adh-DOC (dashed grey line). (b) Histograms show the percentage of T cells coexpressing CD4, CD161, and CD196. (c) The same experiment as in (a) and (b), performed in a 0.4 *μ*m pore polycarbonate transwell system. Upper chamber: CD3^+^ T cells. Lower chamber: DC and DOC. Histograms represent the mean ± SEM of 7 independent experiments. *∗∗* = *p* < 0.01, *∗∗∗* = *p* < 0.001, Bonferroni corrected.

Since recent reports showed that there is a reciprocal relationship between CD4^+^CD25^+^Foxp3^+^ T cells and Th17 cells in their development [28, 29], we asked whether Adh-DOC and Det-DOC showed differential inhibitory capacity during Th17 generation as well as during CD4^+^CD25^+^Foxp3^+^ T cell induction. Since Th17 cells can be identified as CD4^+^CD161^+^CD196^+^ T cells [30, 31], we analyzed the induction of CD4^+^CD161^+^CD196^+^ T cells, after coculture of CD3^+^ T cells with DC alone or incubated either with Det-DOC or with Adh-DOC (Kruskal-Wallis *p* < 0.0001). Coculture of T cells with DC increased the percentage of CD4^+^CD161^+^CD196^+^ T cells, as compared to CD3^+^ T cells alone (Figures 4(a) and 4(b)). Interestingly, the addition of Det-DOC enhanced the generation of CD4^+^CD161^+^CD196^+^ T cells induced by DC, while the addition of Adh-DOC significantly decreased that population (Figures 4(a) and 4(b)). Then, we asked whether the different capacity to modulate the DC-mediated CD4^+^CD161^+^CD196^+^ T cell generation of Det-DOC and Adh-DOC was mediated by cytokine secretion rather than by a cell-to-cell contact-dependent mechanism(s). Therefore, we performed the same assay but after separating CD3^+^ T cells from both DC and DOC with a 0.4 *μ*m pore transwell chamber. As shown in Figure 4(c), when added in the upper chamber of the transwell, CD3^+^ T cells differentiated into CD4^+^CD161^+^CD196^+^ T cells similarly to cell-to-cell contact culture conditions. Therefore, we conclude that hDOC influence the generation of CD4^+^CD161^+^CD196^+^ T cells by DC, by modulating soluble factor(s).

### 3.5. hDOC Modulate the Cytokine Environment

**Figure 5:**
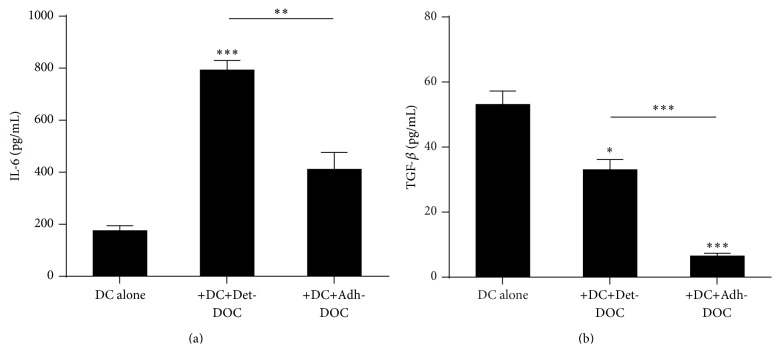
Modulation of the cytokine milieu by hDOC. Quantification of IL-6 (a) and TGF-*β* (b) in the supernatants of DC cultured for 5 days alone or with either Det-DOC or Adh-DOC, at a ratio of DC : hDOC of 1 : 1. Histograms represent the mean ± SEM of the cytokine concentration of 7 independent experiments. *∗* = *p* < 0.05, *∗∗* = *p* < 0.01, and *∗∗∗* = *p* < 0.001, Bonferroni corrected.

To investigate which soluble factor(s) involved in the differentiation of CD4^+^CD161^+^CD196^+^ T cells was regulated by the presence of hDOC, we analyzed the supernatants of the coculture for the presence of TGF-*β* and IL-6, since it has been previously shown that DC can induce Th17 differentiation through the release of IL-6, which acts in concert with TGF-*β* [32]. Our results showed that IL-6 secretion was enhanced in presence of Det-DOC, while it was not influenced by the presence of Adh-DOC (Figure 5(a), Kruskal-Wallis *p* < 0.0001). However, TGF-*β* was decreased in the supernatants of both the cocultures, compared with the supernatant of the DC cultured alone (Figure 5(b), Kruskal-Wallis *p* < 0.0001). To evaluate whether Det-DOC and/or Adh-DOC could modulate the extracellular milieu towards a tolerogenic environment, IL-10 was also tested and no differences were found (Figure S3).

Taken together, these data demonstrate that hDOC can modulate the DC induction of CD4^+^CD161^+^CD196^+^ T cells via IL-6/TGF-*β* regulation. The different capacity of Det-DOC and of Adh-DOC to modulate the induction of the CD4^+^CD161^+^CD196^+^ T cell phenotype may be due to the different balance between IL-6 and TGF-*β* secreted or induced by these cells, a difference that according to our results is generated by the way hDOC are manipulated.

## 4. Discussion

Bone transplantation is the second most common tissue transplantation after blood. The most efficient tissue source would be the autologous bone, but the donor-site morbidity, the inadequate supply, and the problems about size and shape make this source not always feasible. Allografts from cadaveric donors are increasingly used, but the risk of disease transmission and/or immune reaction limit their use [33, 34]. Indeed, allogeneic peptides may be presented by APC, such as DC, on MHC-I and MHC-II molecules and can activate cytotoxic CD8^+^ T cells and CD4^+^ helper T cells. This activation can (i) mediate allograft rejection [35], (ii) dramatically inhibit bone generation [36], and (iii) lead to gradual, long-term immune response [37]. Among the other sources tested to prevent these adverse effects, MSC have been shown to be immunoprivileged, that is, nontargeted by MHC-mismatched immune cells [38], to exert a suppressive effect on the host immune system [39] and to repair bone defects* in vivo* either through infusion or through local implantation [16, 40]. The main limitation for bone repair is the low capacity of engraftment of the* in vitro*-cultured MSC. Since the basic functional unit for the repair of bone defects is the differentiated osteogenic cell (DOC) derived from MSC, it has been suggested that osteogenic differentiation of MSC before implantation might be useful to optimize bone regeneration. In this way, the interval between implantation and subsequent osteogenesis* in situ* might be shortened [18], with a consequent reduction of loss of the implanted cells. However, differentiated osteoprogenitors may be not immunoprivileged and may not have the same suppressive effect on the host immune system as their undifferentiated MSC progenitors.

Here we show for the first time that the immunological properties of hMSC to modulate DC phenotype and functions can persist after* in vitro* differentiation into hDOC. However, our data demonstrate that the capacity of hDOC of modulating recipient DC function depends on different processing protocols and experimental conditions (i.e., the presence or absence of extracellular matrix). In fact, Det-DOC are less potent than Adh-DOC to inhibit the allostimulation of DC in a contact-dependent manner, and this is due to the partial downregulation of the costimulatory molecules expressed on DC. Indeed, Det-DOC do not modify the expression of CD86, while they weakly downregulate CD80. On the other hand, Adh-DOC strongly downregulate CD80 and CD86 on DC, resulting in a more profound inhibition of T cell proliferation. The different capacity of Det-DOC and Adh-DOC to downregulate CD80 and CD86 might also explain why Adh-DOC are more efficient than Det-DOC in inhibiting also the induction of CD4^+^CD25^+^Foxp3^+^ T cells by DC, in a contact-dependent manner. Similarly, Adh-DOC have an opposite effect on DC in the induction of CD4^+^CD161^+^CD196^+^ T cells in comparison with Det-DOC. In fact, Adh-DOC inhibit, while Det-DOC enhance, the capacity of DC to generate CD4^+^CD161^+^CD196^+^ T cells in a cytokine-dependent manner, depending on their capacity to differently modulate the balance between TGF-*β* and IL-6. The same results were obtained also with mature DC (data not shown), suggesting that the capacity of hDOC to modulate DC function is independent from the maturation state of DC.

Overall, here we show that Adh-DOC strongly inhibit some of the main functions of DC, such as the induction of T cell proliferation, the generation of CD4^+^CD25^+^Foxp3^+^, and the generation of CD4^+^CD161^+^CD196^+^ T cells. On the other hand we also show that Det-DOC weakly inhibit the DC capacity of inducing T cell proliferation and of generating CD4^+^CD25^+^Foxp3^+^ T cells, while they enhance the DC capacity of generating CD4^+^CD161^+^CD196^+^ T cells. Thus, the coculture of either Det-DOC or Adh-DOC with DC results in altered APC functions.

## 5. Conclusions

In the present work, we showed that hDOC are able to modulate the function of DC and, therefore, the host immune response. Moreover, by comparing hDOC as cellular suspension (i.e., Det-DOC) to cells implanted on a scaffold (i.e., Adh-DOC), we showed that different manipulation techniques could result in different immunological properties of hDOC. As a consequence, the culture system of hDOC can produce highly different immunological outcome. More conclusive evidence should be gathered through* in vivo* clinical studies. However, our report about different* in vitro* capacity of Det-DOC and Adh-DOC in modulating DC functions offers the rationale to specifically address, at the clinical level, the safety and immunomodulatory capacity of DOC. Indeed, better definition of the most suitable culture system for hDOC preparation may have relevant clinical implications for their implantation in the context of bone repair cell-based tissue-engineering clinical trials.

## Supplementary Material

Table S1: Characteristics of bone marrow and peripheral blood donors. 7 bone marrow donors (BM58, 65, 66, 71, 79, 81 and 82; F:M ratio, 2:5; median age 41) were used to collect MSC. Seven peripheral blood donors (BC41, 42, 52, 55, 60, 61 and 62; F:M ratio, 3:4; median age 47) were used to collect CD3^+^ and CD14^+^ cells.
Figure S1: Evaluation of osteogenic differentiation. hMSC were cultured 2 weeks in DMEM or in osteogenic differentiation medium. Osteogenic differentiation was evaluated by cytological staining through alizarin red staining (A) and by the evaluation of RUNX2 mRNA levels (B). (B) Results are expressed as fold change comparing osteogenic differentiated hDOC with hMSC of 7 independent experiments. ∗∗∗ = p<0.001.
Figure S2: Schema of the experimental design. MSC were differentiated for 14 days to obtain DOC. DOC were kept adherent to the plate (Adh-DOC) or detached, counted and re-seeded (Det-DOC). Adh-DOC and Det-DOC were cultured in complete RPMI 1640 for an overnight, then were put in culture for 5 days with DC and CD3^+^ cells either in a cell-to-cell contact system, or in a transwell system. At day 20 cells and supernatants were collected to be analyzed.
Figure S3: Quantification of IL-10 in the supernatants of the cocultures. IL-10 was evaluated in the supernatants of DC cultured for 5 days alone, or with either Det-DOC or Adh-DOC, at a ratio of DC:hDOC of 1:1. Histograms represent the mean ± SEM of the cytokine concentration of 7 independent experiments.

## References

[1] Pittenger M. F., Mackay A. M., Beck S. C. (1999). Multilineage potential of adult human mesenchymal stem cells. *Science*.

[2] Nauta A. J., Kruisselbrink A. B., Lurvink E., Willemze R., Fibbe W. E. (2006). Mesenchymal stem cells inhibit generation and function of both CD34^+^-derived and monocyte-derived dendritic cells. *The Journal of Immunology*.

[3] Uccelli A., Moretta L., Pistoia V. (2008). Mesenchymal stem cells in health and disease. *Nature Reviews Immunology*.

[4] Tolar J., Le Blanc K., Keating A., Blazar B. R. (2010). Concise review: hitting the right spot with mesenchymal stromal cells. *Stem Cells*.

[5] Le Blanc K., Frassoni F., Ball L. M. (2008). Mesenchymal stem cells for treatment of steroid-resistant, severe, acute graft-versus-host disease: a phase II study. *The Lancet*.

[6] Duijvestein M., Vos A. C. W., Roelofs H. (2010). Autologous bone marrow-derived mesenchymal stromal cell treatment for refractory luminal Crohn's disease: results of a phase I study. *Gut*.

[7] Jiang X.-X., Zhang Y., Liu B. (2005). Human mesenchymal stem cells inhibit differentiation and function of monocyte-derived dendritic cells. *Blood*.

[8] Spaggiari G. M., Abdelrazik H., Becchetti F., Moretta L. (2009). MSCs inhibit monocyte-derived DC maturation and function by selectively interfering with the generation of immature DCs: central role of MSC-derived prostaglandin E_2_. *Blood*.

[9] Banchereau J., Briere F., Caux C. (2000). Immunobiology of dendritic cells. *Annual Review of Immunology*.

[10] Caux C., Vanbervliet B., Massacrier C. (1996). CD34+ hematopoietic progenitors from human cord blood differentiate along two independent dendritic cell pathways in response to GM-CSF+TNF*α*. *Journal of Experimental Medicine*.

[11] Curti A., Fogli M., Ratta M., Tura S., Lemoli R. M. (2001). Stem cell factor and FLT3-ligand are strictly required to sustain the long-term expansion of primitive CD34^+^DR^−^ dendritic cell precursors. *Journal of Immunology*.

[12] Li Y.-P., Paczesny S., Lauret E. (2008). Human mesenchymal stem cells license adult CD34^+^ hemopoietic progenitor cells to differentiate into regulatory dendritic cells through activation of the notch pathway. *The Journal of Immunology*.

[13] Aldinucci A., Rizzetto L., Pieri L. (2010). Inhibition of immune synapse by altered dendritic cell actin distribution: a new pathway of mesenchymal stem cell immune regulation. *Journal of Immunology*.

[14] Le Blanc K., Tammik C., Rosendahl K., Zetterberg E., Olle Ringdén O. (2003). HLA expression and immunologic properties of differentiated and undifferentiated mesenchymal stem cells. *Experimental Hematology*.

[15] Niemeyer P., Kornacker M., Mehlhorn A. (2007). Comparison of immunological properties of bone marrow stromal cells and adipose tissue-derived stem cells before and after osteogenic differentiation in vitro. *Tissue Engineering*.

[16] Quarto R., Mastrogiacomo M., Cancedda R. (2001). Repair of large bone defects with the use of autologous bone marrow stromal cells. *The New England Journal of Medicine*.

[17] Cancedda R., Bianchi G., Derubeis A., Quarto R. (2003). Cell therapy for bone disease: a review of current status. *Stem Cells*.

[18] Tsubota S., Tsuchiya H., Shinokawa Y., Tomita K., Minato H. (1999). Transplantation of osteoblast-like cells to the distracted callus in rabbits. *The Journal of Bone & Joint Surgery—British Volume*.

[19] Sieh S., Lubik A. A., Clements J. A., Nelson C. C., Hutmacher D. W. (2010). Interactions between human osteoblasts and prostate cancer cells in a novel 3D in vitro model. *Organogenesis*.

[20] Ciciarello M., Zini R., Rossi L. (2013). Extracellular purines promote the differentiation of human bone marrow-derived mesenchymal stem cells to the osteogenic and adipogenic lineages. *Stem Cells and Development*.

[21] Trabanelli S., Ocadlíková D., Ciciarello M. (2014). The SOCS3-independent expression of IDO2 supports the homeostatic generation of t regulatory cells by human dendritic cells. *Journal of Immunology*.

[22] Trabanelli S., Očadlíková D., Gulinelli S. (2012). Extracellular ATP exerts opposite effects on activated and regulatory CD4^+^ T cells via purinergic P2 receptor activation. *The Journal of Immunology*.

[23] Curti A., Trabanelli S., Onofri C. (2010). Indoleamine 2,3-dioxygenase-expressing leukemic dendritic cells impair a leukemia-specific immune response by inducing potent T regulatory cells. *Haematologica*.

[24] Jenkins M. K., Johnson J. G. (1993). Molecules involved in T-cell costimulation. *Current Opinion in Immunology*.

[25] Mondino A., Jenkins M. K. (1994). Surface proteins involved in T cell costimulation. *Journal of Leukocyte Biology*.

[26] Finkelman F. D., Lees A., Birnbaum R., Gause W. C., Morris S. C. (1996). Dendritic cells can present antigen in vivo in a tolerogenic or immunogenic fashion. *Journal of Immunology*.

[27] Liang S., Alard P., Zhao Y., Parnell S., Clark S. L., Kosiewicz M. M. (2005). Conversion of CD4^+^ CD25^−^ cells into CD4^+^ CD25^+^ regulatory T cells in vivo requires B7 costimulation, but not the thymus. *The Journal of Experimental Medicine*.

[28] Zhao L., Qiu de K., Ma X. (2010). Th17 cells: the emerging reciprocal partner of regulatory T cells in the liver. *Journal of Digestive Diseases*.

[29] Baban B., Chandler P. R., Sharma M. D. (2009). IDO activates regulatory T cells and blocks their conversion into Th17-like T cells. *The Journal of Immunology*.

[30] Cosmi L., De Palma R., Santarlasci V. (2008). Human interleukin 17-producing cells originate from a CD161^+^CD4^+^ T cell precursor. *Journal of Experimental Medicine*.

[31] Sollazzo D., Trabanelli S., Curti A., Vianelli N., Lemoli R. M., Catani L. (2011). Circulating CD4^+^CD161^+^CD196^+^ Th17 cells are not increased in immune thrombocytopenia. *Haematologica*.

[32] Lee Y. K., Mukasa R., Hatton R. D., Weaver C. T. (2009). Developmental plasticity of Th17 and Treg cells. *Current Opinion in Immunology*.

[33] Reikerås O., Shegarfi H., Naper C., Reinholt F. P., Rolstad B. (2008). Impact of MHC mismatch and freezing on bone graft incorporation: an experimental study in rats. *Journal of Orthopaedic Research*.

[34] Bos G. D., Goldberg V. M., Gordon N. H. (1985). The long-term fate of fresh and frozen orthotopic bone allografts in genetically defined rats. *Clinical Orthopaedics and Related Research*.

[35] Afzali B., Lechler R. I., Hernandez-Fuentes M. P. (2007). Allorecognition and the alloresponse: clinical implications. *Tissue Antigens*.

[36] Trombetta E. S., Mellman I. (2005). Cell biology of antigen processing in vitro and in vivo. *Annual Review of Immunology*.

[37] Shegarfi H., Reikeras O. (2009). Review article: bone transplantation and immune response. *Journal of Orthopaedic Surgery*.

[38] Krampera M., Glennie S., Dyson J. (2003). Bone marrow mesenchymal stem cells inhibit the response of naive and memory antigen-specific T cells to their cognate peptide. *Blood*.

[39] Aggarwal S., Pittenger M. F. (2005). Human mesenchymal stem cells modulate allogeneic immune cell responses. *Blood*.

[40] Wilson T., Stark C., Holmbom J. (2010). Fate of bonemarrow-derived stromal cells after intraperitoneal infusion or implantation into femoral bone defects in the host animal. *Journal of Tissue Engineering*.

