# Supplementary material for: The Human Mesenchymal Stromal Cell-Derived Osteocyte Capacity to Modulate Dendritic Cell Functions Is Strictly Dependent on the Culture System
Source: J Immunol Res. 2015 Jul 12;2015:526195. doi: 10.1155/2015/526195 (PMC4515297; doi:10.1155/2015/526195)
Supplement: Supplementary file 1 — Table S1: Characteristics of bone marrow and peripheral blood donors. 7 bone marrow donors (BM58, 65, 66, 71, 79, 81 and 82; F:M ratio, 2:5; median age 41) were used to collect MSC. Seven peripheral blood donors (BC41, 42, 52, 55, 60, 61 and 62; F:M ratio, 3:4; median age 47) were used to collect CD3+ and CD14+ cells. Figure S1: Evaluation of osteogenic differentiation. hMSC were cultured 2 weeks in DMEM or in osteogenic differentiation medium. Osteogenic differentiation was evaluated by cytological staining through alizarin red staining (A) and by the evaluation of RUNX2 mRNA levels (B). (B) Results are expressed as fold change comparing osteogenic differentiated hDOC with hMSC of 7 independent experiments. ∗∗∗ = p<0.001. Figure S2: Schema of the experimental design. MSC were differentiated for 14 days to obtain DOC. DOC were kept adherent to the plate (Adh-DOC) or detached, counted and re-seeded (Det-DOC). Adh-DOC and Det-DOC were cultured in complete RPMI 1640 for an overnight, then were put in culture for 5 days with DC and CD3+ cells either in a cell-to-cell contact system, or in a transwell system. At day 20 cells and supernatants were collected to be analyzed. Figure S3: Quantification of IL-10 in the supernatants of the cocultures. IL-10 was evaluated in the supernatants of DC cultured for 5 days alone, or with either Det-DOC or Adh-DOC, at a ratio of DC:hDOC of 1:1. Histograms represent the mean ± SEM of the cytokine concentration of 7 independent experiments. [file 526195.f1.pdf]

Donor	Material	Isolated Cells	Condition	Sex	Age
BM58	bone marrow	MSC	healthy	M	41
BM65	bone marrow	MSC	healthy	F	32
BM66	bone marrow	MSC	healthy	M	44
BM71	bone marrow	MSC	healthy	F	62
BM79	bone marrow	MSC	healthy	M	22
BM81	bone marrow	MSC	healthy	M	19
BM82	bone marrow	MSC	healthy	M	43
BC41	peripheral blood	$CD3^+/CD14^+$ cells	healthy	M	45
BC42	peripheral blood	$CD3^+/CD14^+$ cells	healthy	F	52
BC52	peripheral blood	$CD3^+/CD14^+$ cells	healthy	F	26
BC55	peripheral blood	$CD3^+/CD14^+$ cells	healthy	M	31
BC60	peripheral blood	$CD3^+/CD14^+$ cells	healthy	M	47
BC61	peripheral blood	$CD3^+/CD14^+$ cells	healthy	F	60
BC62	peripheral blood	$CD3^+/CD14^+$ cells	healthy	M	58

Table S1. Characteristics of bone marrow and peripheral blood donors. 7 bone marrow donors (BM58, 65, 66, 71, 79, 81 and 82; F:M ratio, 2:5; median age 41) were used to collect MSC. Seven peripheral blood donors (BC41, 42, 52, 55, 60, 61 and 62; F:M ratio, 3:4; median age 47) were used to collect $CD3^+$ and $CD14^+$ cells.

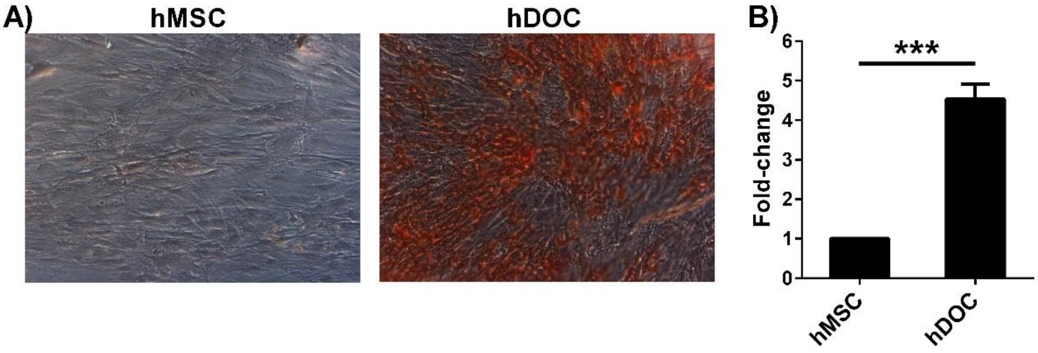

Figure S1. Evaluation of osteogenic differentiation. hMSC were cultured 2 weeks in DMEM or in osteogenic differentiation medium. Osteogenic differentiation was evaluated by cytological staining through alizarin red staining (A) and by the evaluation of RUNX2 mRNA levels (B). (B) Results are expressed as fold change comparing osteogenic differentiated hDOC with hMSC of 7 independent experiments. *** = p<0.001.

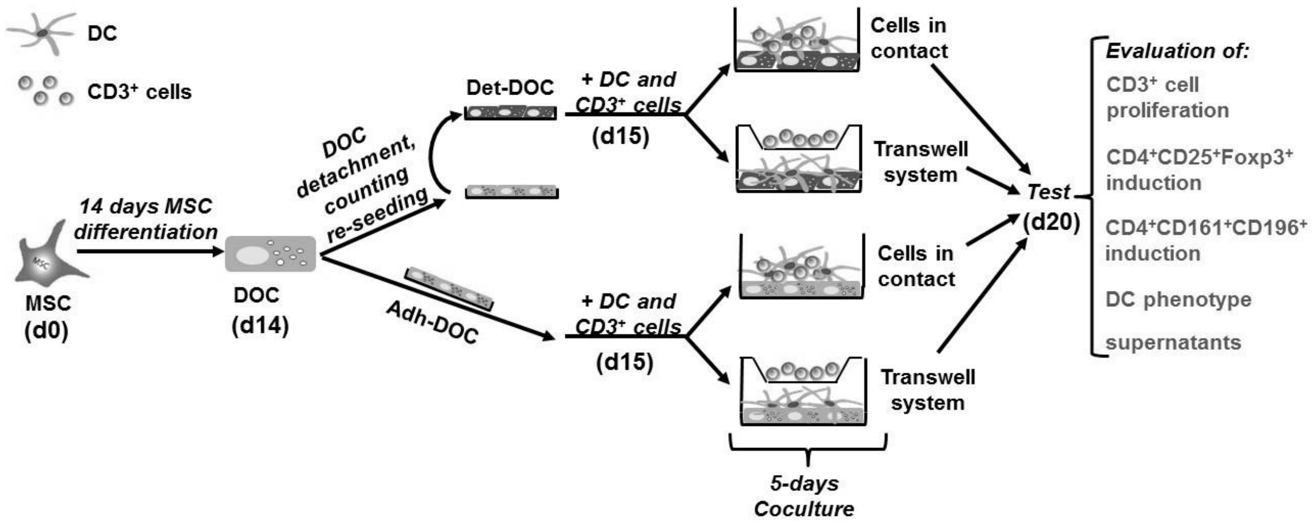

Figure S2. Schema of the experimental design. MSC were differentiated for 14 days to obtain DOC. DOC were kept adherent to the plate (Adh-DOC) or detached, counted and re-seeded (Det-DOC). Adh-DOC and Det-DOC were cultured in complete RPMI 1640 for an overnight, then were put in culture for 5 days with DC and $CD3^+$ cells either in a cell-to-cell contact system, or in a transwell system. At day 20 cells and supernatants were collected to be analyzed.

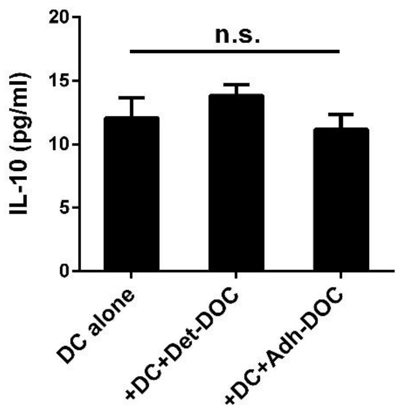

Figure S3. Quantification of IL-10 in the supernatants of the cocultures. IL-10 was evaluated in the supernatants of DC cultured for 5 days alone, or with either Det-DOC or Adh-DOC, at a ratio of DC:hDOC of 1:1. Histograms represent the mean ± SEM of the cytokine concentration of 7 independent experiments.
